# Live process modeling with the BPMN Sketch Miner

**DOI:** 10.1007/s10270-022-01009-w

**Published:** 2022-06-28

**Authors:** Ana Ivanchikj, Souhaila Serbout, Cesare Pautasso

**Affiliations:** Software Institute, USI, Lugano, Switzerland

**Keywords:** Business Process Model and Notation (BPMN), Process mining, Domain-specific languages, Performance evaluation

## Abstract

BPMN Sketch Miner is a modeling environment for generating visual business process models starting from constrained natural language textual input. Its purpose is to support business process modelers who need to rapidly sketch visual BPMN models during interviews and design workshops, where participants should not only provide input but also give feedback on whether the sketched visual model represents accurately what has been described during the discussion. In this article, we present a detailed description of the BPMN Sketch Miner design decisions and list the different control flow patterns supported by the current version of its textual DSL. We also summarize the user study and survey results originally published in MODELS 2020 concerning the tool usability and learnability and present a new performance evaluation regarding the visual model generation pipeline under actual usage conditions. The goal is to determine whether it can support a rapid model editing cycle, with live synchronization between the textual description and the visual model. This study is based on a benchmark including a large number of models (1350 models) exported by users of the tool during the year 2020. The main results indicate that the performance is sufficient for a smooth live modeling user experience and that the end-to-end execution time of the text-to-model-to-visual pipeline grows linearly with the model size, up to the largest models (with 195 lines of textual description) found in the benchmark workload.

## Introduction

The Business Process Model and Notation (BPMN) standard [35] facilitates the communication and knowledge sharing between domain experts, process participants, and business analysts due to the standardized language [68] and its graphical visual notation [51] for modeling business processes [66]. Most BPMN modeling editors support the design of business process models by dragging, dropping and connecting visual elements. However, experience with using UML at Ericsson [44] has shown that “when a DSL is large, covers a wide aspect and has different types of users, having one notation often does not suit the needs of all its users.” Thus, following existing text-based modeling tools (e.g., PlantBPMN [16], PlantUML^1^ and its PlantText^2^ environment, ZenUML,^3^ WebsequenceDiagrams,^4^ Textografo^5^), which generate visual models from textual descriptions, we have designed a tool which supports a textual notation for BPMN. In this article, we present the design and evaluation of the tool, which we called BPMN Sketch Miner.^6^ It is a proof-of-concept tool for rapidly generating BPMN models from descriptions specified with a textual domain-specific language (DSL).

The aim of the tool is to speed up the iteration cycles of requirements gathering leading to the initial sketch of a process model, with a language mimicking structured notes taken during participant interviews [53] and design workshops [8, 42]. To accomplish the goal, the tool augments textual modeling with process mining, thus reducing the complexity and the number of keywords of the textual DSL. Its modeling environment replaces the complex stencil palette usually found in graphical editors with a simple text editor. Textual descriptions are transformed into diagrams, which correctly use the BPMN visual syntax, simultaneously while the modeler is typing them. The main language design challenge consists of the trade-off between usability, learnability, and expressiveness of the textual DSL. While BPMN is a rich notation with hundreds of constructs, our DSL focuses on a subset of the notation [69] with the intention to support an iterative model refinement process, where the initial model is obtained quickly from its constrained natural language description. Basic type annotation keywords need to be learned only if it becomes necessary to classify model elements during a second refinement step. At this point, the model can also be exported so that its refinement can continue using traditional standard-compliant BPMN editors [12]. In other words, we see text-based modeling as complementary to graphical editors, and particularly useful to quickly get started with an initial sketch.

In general, we present a model-driven engineering use case, where process models are inferred from process instances, but also explicitly described where such process mining step would fall short of bringing the proposed textual process modeling notation closer to the practitioners’ domain.

The main contributions of this article are: (1) the design of a tool which needs to balance the trade-off between the use of textual modeling and process mining for quickly creating valid visual BPMN models and (2) the evaluation of the tool with respect to its usability, learnability, DSL expressiveness, as well as its performance under real-world usage conditions.

The evaluation of the usability and learnability of the tool was originally published in [31].^7^ We conducted a user study and surveys [33] involving students having no prior knowledge of the BPMN language, as well as industry analysts, with advanced BPMN knowledge, who typically enjoy working with “quick modeling” shortcuts as they need to, as mentioned by one of them, “represent the content of their processes without having to fiddle with the graphical layout.” The results of this evaluation are summarized in this article, which also includes: (a) additional answers to the SUS survey questionnaire with respect to the ones presented in MODELS 2020 and (b) qualitative feedback from users about the benefits and real world applicability of the tool (Table 4).

In terms of DSL expressiveness, this article presents how the textual language has been further extended to support additional BPMN constructs, such as data and data store objects as well as interrupting and non-interrupting boundary events. This has resulted with greater coverage of the BPMN standard metamodel, as shown using workflow patterns in Sect. 2.2.3.

The performance evaluation is focused on determining whether the tool can support a live modeling cycle with minimal delay in the synchronization of the visual BPMN diagram obtained from the textual description. More in detail, we are interested to detect the presence of bottlenecks in the model generation pipeline, which is present in many of the above-mentioned text-to-graphical modeling tools. This helps to determine whether and where further performance optimization investments should be directed by the model-driven engineering research community. Is this bottleneck specific to the process mining and model inference step? Or does it lie in the model transformation, rendering and layout steps, which also appear in other text-to-graphical modeling tools as well?

To answer these questions, we have performed an extensive performance evaluation based on workloads obtained from real-world usage. The tool implementation has been instrumented to obtain a detailed performance profile of the textual-to-graphical model pipeline execution, and metrics for characterizing the size of the input and output models have been defined.

The main result is that within the tool operating range, the tool performance is sufficient to keep the visual and textual representation synchronized as the user edits the text: Executing the text-to-model-to-visual-diagram pipeline took 77 ms on average and 513 ms in the worst case. Also within the range of model sizes of the given workload (up to 195 lines of text resulting in models with up to 160 nodes and 164 edges), the performance scales linearly with different process size metrics. The BPMN construct which has the most impact on performance is swimlanes, which stress the automatic layout, by far the most expensive of the tool pipeline stages.

The rest of this article is organized as follows: In Sect. 2, we describe the design of the BPMN Sketch Miner and its textual DSL, while in Sect. 3 we summarize the results of the usability and learnability evaluation. We present the results from the conducted performance measurements in Sect. 4. We provide an overview of the related work in Sect. 5 while drawing some conclusions in Sect. 6 before outlining our plans for future work in Sect. 7.

## BPMN Sketch miner design

**Fig. 1:**
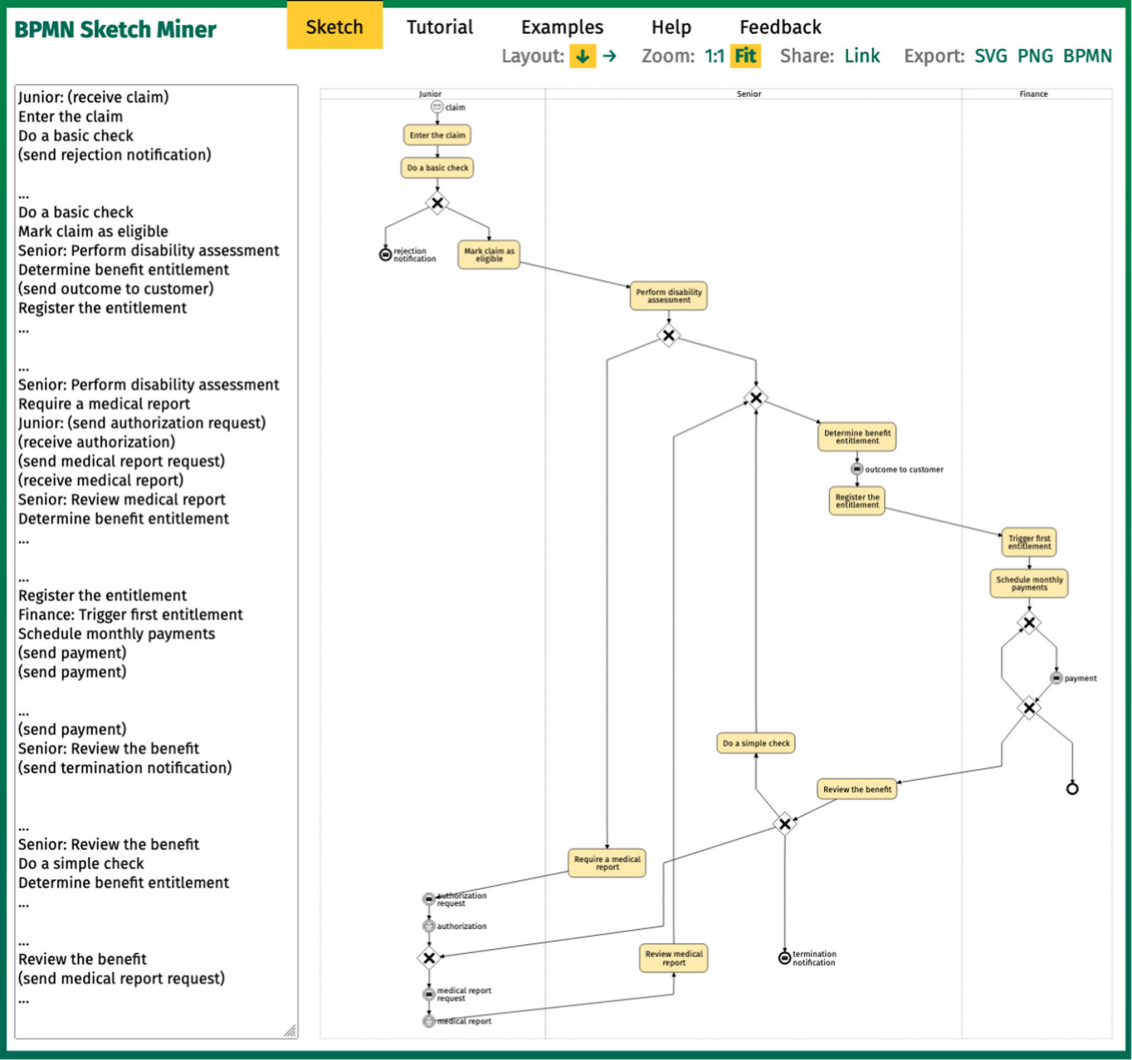
BPMN Sketch Miner with the textual and visual BPMN representation of the process modeled during the user study of [31]. To make it easier to read, click here for opening the diagram in the tool

The design of BPMN Sketch Miner (Fig. 1) is driven by the attempt to trade off the expressiveness against the learnability of the textual DSL, without sacrificing the efficiency with which the tool can be used to produce BPMN diagrams.

### Live modeling environment

We have identified two different contexts where the BPMN Sketch Miner can be used: (1) during co-located or remote requirements elicitation meetings between domain experts and business analysts and (2) in classrooms while teaching BPMN. In the first context, there can be two usage scenarios: the more common one, where the business analysts interview the domain experts while creating the BPMN model, or the one where domain experts are empowered to participate in the creation of the BPMN model themselves. That said, the goal of BPMN Sketch Miner is to streamline the rapid model creation for obtaining feedback from domain experts and process participants, and to facilitate learning BPMN and process modeling. Studies have shown that starting with textual input (e.g., activity tables, written use-case scenarios) and then abstracting the information using visual models improves process understanding for people who are not process modeling experts [5, 11]. Thus, given the goal of our tool, and the contexts in which its use is envisioned, our first design decision was to use textual input for the creation of visual models to be represented in BPMN. The second design decision, which is the focus of this paper’s performance evaluation, was to introduce a live modeling environment, where the BPMN model is produced in real time as the textual description is typed. The third design decision was to deliver it as a web-based tool, thus avoiding the need for software installation to get started with a modeling session.

The main flow of interaction with the tool involves the following steps:

1. Open a web link to go directly into the modeling environment (no user registration or authentication is required).
2. To edit an existing model, use the provided link which embeds its textual description. The link can be easily shared in an email message, chat room, or also embedded in documentation. For example, clicking on any BPMN diagram shown in this paper will open the diagram in the tool.
3. As the textual description is edited, the BPMN diagram is immediately updated (no need to click a submit or refresh button).
4. At the end of the session, export the generated BPMN model in SVG, PNG, or a standard-compliant BPMN XMI file formats so that it can be displayed or further refined in any compatible tool.

### BPMN as a textual domain-specific language

The main feature of the BPMN Sketch Miner is its textual DSL, by means of which the user can enter a textual description of a process. The purpose of the textual language is to provide a simple notation for easy and rapid [23] representation of a process by enumerating execution traces of its instances (e.g., while taking notes describing concrete examples during an interview) from which valid BPMN sketches (i.e., non-executable models [3]) can be obtained.

The main design constraint for the DSL is that it should reflect the largest possible subset of BPMN (a rather large and complex visual notation [69]) while using a limited number of textual constructs, which should be easy to learn and remember. Unlike the graphical syntax, the textual one is characterized by its mono-dimensional structure [22]. This constraint makes it an adequate choice for representing sequential business processes but makes it challenging to use plain text to represent control flow graphs of arbitrary structures like the ones which can be visualized in BPMN. Like first proposed in [30], we address this challenge by using process mining [62] to reconstruct a model of the process control flow graph from a set of sequential execution traces, which can be easily written in plain text. This also makes it possible to target domain experts being interviewed during requirements elicitation who have no or limited BPMN knowledge. Following Karsai et al. [36]’s advice, the textual DSL used in the tool represents ordered lists of tasks and events that are envisioned to be written as the process participants enumerate their activities, or as BPMN students read a given process description they are supposed to model. These lists are then used as input traces by a process mining algorithm.

**Table 1:** BPMN constructs supported by the BPMN Sketch Miner

BPMN construct	Derived from mining	Explicitly modeled	DSL construct
Tasks		$\checkmark$	State each task name in a new line
Task type		$\checkmark$	Use the “manual,” “user,” “script,” “rule,” “service,” “send,” “receive” keywords
Events	$\checkmark$	$\checkmark$	Use “( )” to indicate an event. Whether it is a start, intermediate, boundary or end event is automatically inferred.
Event type		$\checkmark$	Use the “time,” “error,” “receive,” “send,” “notify,” “publish,” “escalate,” “terminate” keywords
Link events	$\checkmark$		Derived from the lack of a matching fragment
Boundary event type		$\checkmark$	Use the “deadline,” “exception,” “received,” “escalated” keywords, use “(( ))” to indicate non-interrupting boundary event
Exclusive gateway split/merge	$\checkmark$		Repeat the last common task before the split
Labeled exclusive gateway split		$\checkmark$	Use “?” after the name of the label
Loops	$\checkmark$		Repeat the tasks in the loop
Sequence flow	$\checkmark$		State tasks in the order in which they happen
Conditional flow	$\checkmark$		State the condition following the gateway label
Message flow	$\checkmark$		Use matching names for the throw and catch events
Event-based gateway	$\checkmark$		The last common task before the split has to be followed by events
Parallel AND gateway		$\checkmark$	Separate the parallel tasks with “$\mid$”
Lanes		$\checkmark$	State the name of the lane followed by “:” and a task
Pools	$\checkmark$		Lanes become pools in case of message exchange
Data objects		$\checkmark$	Use “[ ]” to delimit a data object or the state of a data object
Data store		$\checkmark$	Use “db [ ]” keyword to delimit a data store, text can be included in “[ ]” to name the data store.
Text annotation		$\checkmark$	Use “//” before the task to which the text annotation is to be attached

#### Design decisions

Based on continuous formative evaluation with test users, including BPMN consultants, experts, and trainers, we have extended the textual DSL, which now supports the BPMN constructs shown in Table 1. The most important design decisions involve:

- annotating the name of the role followed by “:”, to speed up annotating tasks or events with their roles. The role is applied to all tasks following the annotation until another role is declared. The roles are automatically mapped to swimlanes or pools depending on the presence or absence of handovers and message exchange between them.
- events are distinguished from tasks because they are entered in round parenthesis referring to their round visual shape. The mining algorithm determines automatically whether they are *start*, *intermediate*, or *end* events, depending on whether there are stated tasks preceding/following the event in question.
- boundary events use their own type annotations, which are related to the corresponding event type. For example, the start/intermediate receive or intermediate/final escalate event corresponds to the received or escalated boundary events. Likewise, the timer or error events correspond to the deadline or exception boundary events. This should make it easier to remember the event type keywords without having to specify one more keyword to distinguish boundary events such as boundary timer. Non-interrupting boundary events are entered in double round parenthesis referring again to their visual shape.
- data objects are detected because square brackets are found within the corresponding line. We chose to use square brackets as they are commonly used to annotate the state of a data object [47]. As databases are commonly used for permanent data storage, we use the keyword db before the square brackets to depict a data store.
- tasks and events can be annotated by prefixing them with a type. This addresses the requirement of business analysts who need to refine their model after the initial sketch. Stating event types and task types requires the use of keywords, which attempt to match how those constructs are named in BPMN.
- exclusive split gateways can be annotated with a label, specified as a question (i.e., a line ending with ?) in the text. As exclusive gateways denote decisions, the line after the question represents the chosen alternative and becomes the expression associated with the outgoing conditional flow of the gateway.
- we intentionally decided not to mine parallel gateways as it would require manually entering multiple traces including different permutations of the same set of tasks, which early adopters considered too much effort. Instead, parallel tasks are simply declared on the same line by separating them with “!|!”.

The design of the BPMN Sketch Miner’s textual DSL attempts to draw the line between the usefulness of mining algorithms to infer BPMN constructs implicitly embedded into the textual description vs. explicitly stating a construct, as evident in Table 1.

Our goal is, whenever possible, to reduce the cognitive effort of the users [68] by not requiring them to explicitly state BPMN constructs. For example, as event-based gateways can be deduced easily by analyzing traces in which the same task is followed by different events, we do not require the user to explicitly include such gateways in the textual description. The same applies to message flows which can be inferred by detecting a matching message name for a pair of send and receive tasks or message events. Likewise, lanes are automatically clustered into pools based on the presence of message flow or sequence flow handovers.

#### Language syntax

The textual DSL concrete syntax is meant to be closer to natural language than to common textual programming languages, which are based on control structures, blocks, and the use of keywords. The textual DSL’s context-free grammar is expressed in the following EBNF specification:

- $$\langle text\rangle $$ $${:}{:}{=}$$ ($$\langle trace\rangle $$ ’EOL’)$$^+$$ ’EOF’;
- $$\langle trace\rangle $$ $${:}{:}{=}$$ $$\langle dots\rangle $$ ($$\langle line\rangle $$ ’EOL’ $$\langle dots\rangle $$)$$^+$$;
- $$\langle dots\rangle $$ $${:}{:}{=}$$ [’...’ ’EOL’] ;
- $$\langle line\rangle $$ $${:}{:}{=}$$ $$\langle parallel\rangle $$ | $$\langle annotation\rangle $$ | $$\langle comment\rangle $$ ;
- $\langle comment\rangle$ ${:}{:}{=}$ ’///’ (any character until EOL)$^*$ ;
- $$\langle annotation\rangle $$ $${:}{:}{=}$$ ’//’ $$\langle label\rangle $$ ;
- $$\langle parallel\rangle $$ $${:}{:}{=}$$ $$\langle element\rangle $$ (’|’ $$\langle element\rangle $$ )$$^*$$;
- $$\langle element\rangle $$ $${:}{:}{=}$$ [$$\langle role\rangle $$] ($$\langle task\rangle $$ | $$\langle event\rangle $$ | $$\langle XOR label\rangle $$ | $$\langle data\rangle $$) | $$\langle role\rangle $$;
- $$\langle role\rangle $$ $${:}{:}{=}$$ $$\langle label\rangle $$ ’:’ ;
- $$\langle XOR label\rangle $$ $${:}{:}{=}$$ $$\langle label\rangle $$ ’?’ ;
- $$\langle task\rangle $$ $${:}{:}{=}$$ [$$\langle ttype\rangle $$] $$\langle label\rangle $$;
- $$\langle event\rangle $$ $${:}{:}{=}$$ ’(’ [$$\langle etype\rangle $$ | $$\langle ibetype\rangle $$] $$\langle label\rangle $$ ’)’ | ’((’ $$\langle betype\rangle $$ $$\langle label\rangle $$ ’))’;
- $\langle ttype\rangle$ ${:}{:}{=}$ (’send’ | ’receive’ | ’user’ | ’manual’ | ’service’ | ’script’ | ’rule’ ) ;
- $\langle etype\rangle$ ${:}{:}{=}$ (’start’ | ’finish’ | ’timer’| ’send’ | ’receive’ | ’publish’ | ’notify’ | ’error’ | ’escalate’ | ’terminate’ ) ;
- $\langle ibetype\rangle$ ${:}{:}{=}$ (’deadline’ | ’exception’ | ’received’ | ’escalated’ ) ;
- $\langle betype\rangle$ ${:}{:}{=}$ (’deadline’ | ’received’ | ’escalated’ ) ;
- $$\langle data\rangle $$ $${:}{:}{=}$$ ’[’ [$$\langle dtype\rangle $$] $$\langle label\rangle $$ ’]’ | [$$\langle dtype\rangle $$] $$\langle label\rangle $$ ’[’ $$\langle label_state\rangle $$ ’]’ ;
- $$\langle dtype\rangle $$ $${:}{:}{=}$$ (’db’) ;
- $$\langle label_state\rangle $$ $${:}{:}{=}$$ $$\langle label\rangle $$ ;
- $\langle label\rangle$ ${:}{:}{=}$ any valid BPMN element label;

The syntax first breaks down the textual description as a set of traces: sequences of lines, which may or may not be fragments, i.e., include three dots. Then, lines are classified as comment lines (which are ignored), annotations, or elements.

Annotation labels are implicitly associated with the element found on the following line, unless they are on the last line of a sequence when they are associated with the preceding element. Multiple lines with annotations are merged together in the same visual element.

While each line of text usually represents one model element, they can actually represent multiple elements only if these elements are part of a parallel flow (indicated by the pipe !|! character). Otherwise, the language follows the rule: Each element is declared on a different line.

Elements can be placed into pools or swimlanes by preceding them by a role annotation (labels followed by the colon !:! character). Stand-alone role annotation (not followed by any element on the same line) provides the default role for the elements which are first found on the next lines, unless their role is overridden by a role annotation found on the same line.

Elements can be tasks, events, XOR gateway labels, or data objects. Different non-task elements are detected by using different kinds of punctuation or parenthesis: (round) for events, [square] for data objects, and question marks? for XOR gateway labels.

Tasks and events can be further classified by including a keyword representing their type as the first word of their label. This will be omitted from the label and transformed into the corresponding visual icon.

While events are automatically turned into start, intermediate, or end event, different event-type keywords are used to place them on the task boundary (*ibetype* and *betype*). Since boundary events can be interrupting or non-interrupting, we introduce an additional set of round parenthesis to distinguish them. While events, as tasks, can also be left untyped, boundary events require a specific type annotation.

While the events should be placed within single or double round parenthesis, data objects are listed within square parenthesis, but can also follow the common practice of only enclosing the state of the data object within square parenthesis. In this case, the square parenthesis will be kept and displayed as part of the data object label in the diagram. Data objects can also be annotated with an optional type, in this case, used to turn them into data storage elements.

#### Control flow patterns

**Table 2:**
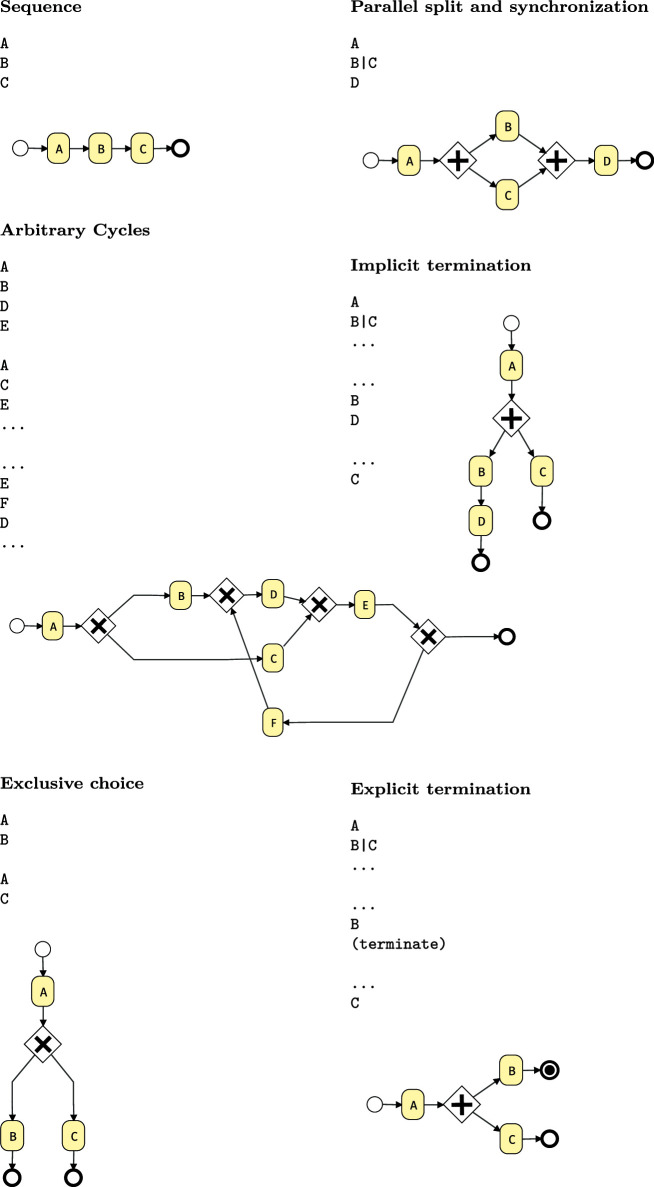
Control flow patterns [63] supported by the BPMN Sketch Miner DSL. To make it easier to read, click on the text link will open the diagram in the tool. Arbitrary Cycles, Exclusive choice, Parallel split and synchronization, Implicit termination, Explicit termination

**Table 3:**
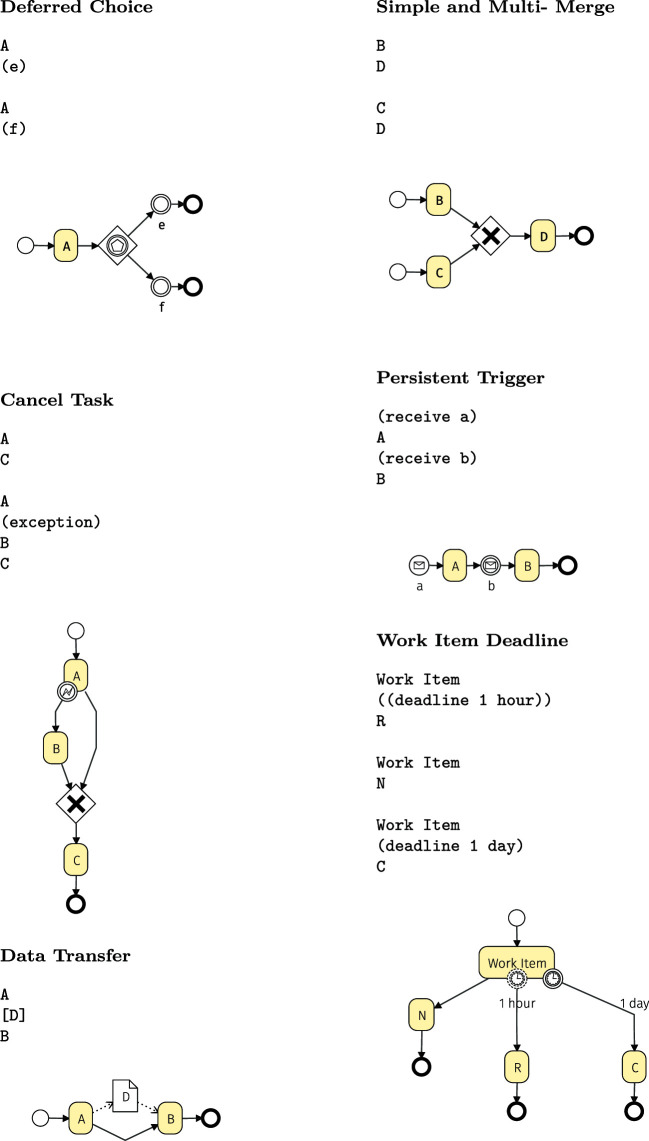
Control flow patterns [63] supported by the BPMN Sketch Miner DSL. To make it easier to read, click on the text link will open the diagram in the tool. Deferred Choice,Cancel Task, Data Transfer, Simple and Multi-Merge, Persistent Trigger, Work Item Deadline (Continued)

While BPMN support for workflow patterns is well known [37, 56, 67], in Tables 2 and 3 we show a few abstract usage examples to illustrate to which extent the BPMN Sketch Miner textual DSL supports a number of important workflow patterns. The tables showcase how these frequently used modeling patterns can be expressed with the textual DSL.

**Fig. 2:**
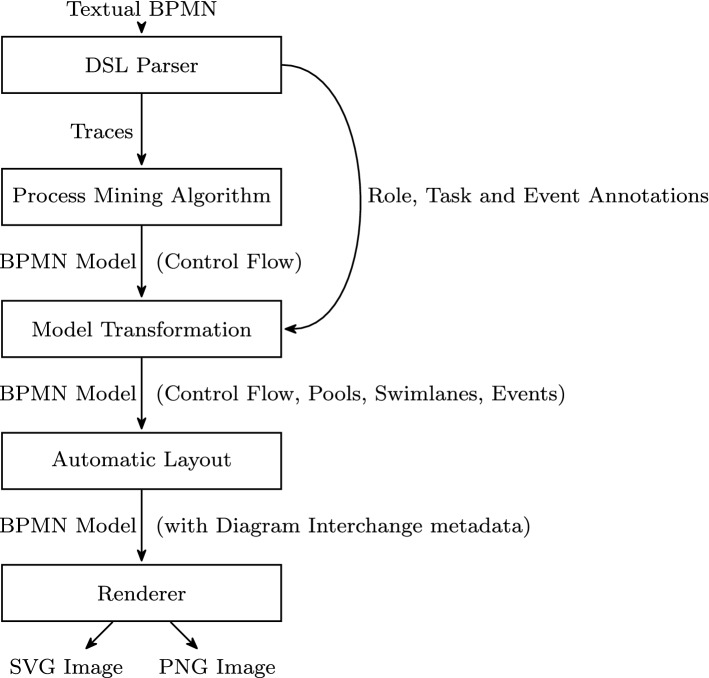
Model generation and transformation pipeline

### Model generation pipeline

Using textual input to generate visual BPMN models requires multiple model generation and transformation steps (Fig. 2). The initial control flow model is generated using a process mining algorithm applied to traces that are extracted from the textual input. The textual annotation of roles, task, and event types is part of the textual modeling and thus is not fed into the mining algorithm, but used during a second stage as follows: First, the nodes of the control flow graph are transformed into tasks, data objects, or events (according to the type annotations found in the original description). Then, the role annotations are used to place the elements in the corresponding swimlanes.

This nonlinear pipeline structure makes it possible to merge the result of the process mining stage with the additional modeling information provided by the user as part of the original input.

While this would already be sufficient for obtaining a valid BPMN process model, in order to make the result visible, the model needs to be further augmented with additional diagram interchange metadata. This is produced by a hierarchical layout algorithm [17, 20], which has been tailored to consider idioms of the notation and can produce both vertical and horizontal layouts.

**Table 4:** Qualitative feedback from anonymous users

Feedback	Date
I like the idea of generating BPMN from text. I am just afraid of having to learn a new semantic from scratch	2020-02-19
Great BPMN Tool.	2020-03-05
Great idea, easy to use. Amazing integration of BPMN objects	2020-03-11
Fantastic tool, having daily use of it. Link feature is impressive	2020-04-07
Once Upon A time John O’Connell began Staffware and invented BPM. it was really challenging because you had to build workflows in a text file. Then Staffware invented iProcess modeler which set the BPM world in motion. Graphically building workflows that did real work. Intuitive, awesome for businesses to build workflows and IT to implement them. Then everyone saw it was good and made their own. Then everyone wanted more capabilities and a standard for models BPMN and BPEL was born. The modelers grew too complicated for business owners to easily build workflows, so it was back to writing specs and having IT build stuff. Something had to be done. BPMN-Sketch-Miner came along and fixed the problem by making it quick and easy to build a workflow by typing text again, and generating visual workflows	2020-09-06
I really like your program and the whitepaper that started it. I used it in the past during workshops to quickly note down a prototype of a process	2021-03-09
I found very interesting this software, I would like to see improvements about efficiency of visualization of large projects	2021-03-09
I absolutely love the fact that one can actually come out with a process diagram during a process discovery or requirement gathering meeting. Also diagram changes are fairly easy to implement, and the alignment is completely automated. I believe if the general population sees this benefit instead of the necessity to type (which to many may sound scary in the beginning) I see a great potential for the Sketch Miner. it really allows us to focus on the meaning, not the “drawing”	2021-03-15
I really liked a lot the tool. It is a great idea to be able to describe a process only in textual form and let the tool automatically create the graphical diagrams. I always found very boring to draw diagrams and reorganize the elements	2021-04-27
I am blind. So for me, the accessibility is the most important part. Basically, the text input feature is very useful for me	2021-10-29
I found BPMN Sketch Miner by accident when I was trying to find out how to do BPMN modeling with either mermaid.js or PlantUML and in a forum I read someone’s post saying you could try this. I have been experimenting with it ever since and will be implementing it in a workshop next week where I think it will save a lot of time. Thank you so much! I am also reading your leanpub business-process-management book that is filling in a load of gaps in my understanding. When I found that I could export from the tool directly to Sparx Enterprise Architect, I thought it was Christmas, again... and it’s only January! I am loving this tool already, thank you!	2022-01-18
I am about to conduct some ongoing workshops to help with a business and IT system integration of 2 large companies. I see sketch miner helping to rapidly help us to discover and explore, to create a shared language and to find the integrations faster and more efficiently	2022-01-18
Absolutely loving this software, thank you! This is going to save innumerable hours on my project! I am telling colleagues about it... It should be renamed to ’Gold Mine!’ because that’s what I think I have found	2022-01-22

The result can be exported as standard BPMN2.0/XML files, but also rendered as vector or bitmap images, using the dagre-d3 library^8^ to display the result.

## Usability and learnability evaluation summary

In this section, we summarize the main findings of the usability and learnability evaluation presented in [31]. In addition, complementing those quantitative results, Table 4 highlights some of the qualitative feedback about the tool which was spontaneously and anonymously submitted by its users over the past two years.

The usability and learnability were evaluated with different survey questionnaires, which were answered after users had an opportunity to practice modeling processes with the tool. As part of the user populations, we included 21 students which were not familiar with the BPMN modeling notation as well as 9 industry experts.

### Model understanding and accuracy

Given a specific, non-trivial process modeling scenario (Fig. 1), the students participating in the study were able to obtain correct models (average correctness of 87%) in a limited time (half completed the task between 30 min and 1 h). The study participants grasped well how to enumerate different process instances by stating them as lists of tasks, how to distinguish tasks from events as well as how to provide the correct decision (XOR) gateway labels. While they were not taught how to draw BPMN constructs in order to model the process, they found the automatically generated visual model beneficial for their understanding of the process.

### System usability scale

**Fig. 3:**
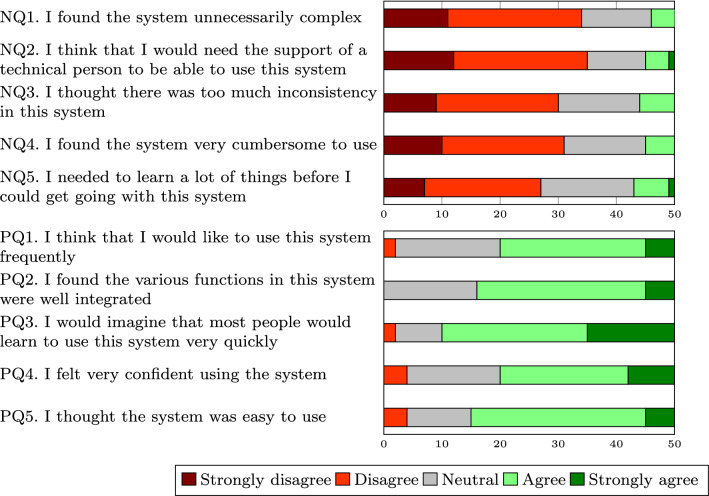
System usability scale (SUS) survey results

**Fig. 4:**
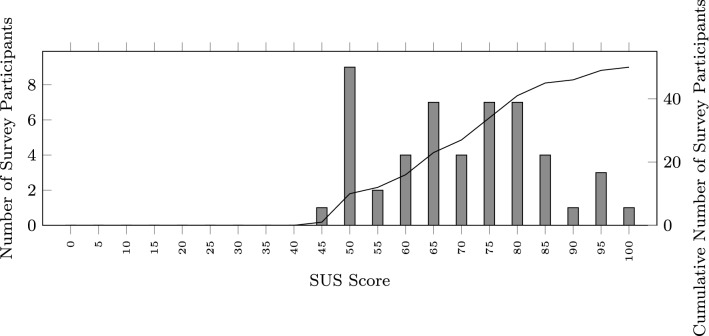
SUS score histogram

The results presented in detail in [31] already indicated an acceptable level of usability (based on the answers to the standard SUS survey collected from 30 participants). In Fig. 3, we show the SUS survey results including the answers of an additional 20 participants. The combined result confirms the usability score of 69 as an average of all the answers, with a 95% confidence interval of 65–73. The histogram of the distribution of the SUS scores is shown in Fig. 4.

Although in general, higher scores indicate better usability, the thresholds used to interpret the score differ in different studies. In [45], they state that a score below 64 is unacceptable, above 85 is excellent and everything in between is acceptable.

### Efficiency and learnability

The majority of users agreed with the fact that using the tool does not require much cognitive effort. Also, most users agree with the fact that they can type faster than they can model the process using a graphical editor.

Only two out of 50 respondents of the SUS survey disagreed with the statement that most people would learn to use this system very quickly. Seven respondents realized that they would need to learn a lot of things before they could get going with the system.

To help with the on-boarding and engagement of the users of the tool, in addition to the DSL documentation, we have also added tutorials, to get the users started, and examples to show-case different modeling challenges. These were found to be either very or extremely helpful by the survey respondents.

## Performance evaluation

The goal of the performance evaluation is to determine whether the performance of the tool is good enough to support the live modeling cycle, where the visual BPMN model is refreshed as users type its textual description.

The live modeling requirement places a clear target on the tool performance: The visual model should be updated as the user types its textual description. This is particularly important for model sketching tools, whereby an initial model should be immediately obtained as the user takes notes, for example, during a modeling workshop. Any delay due to: (1) users having to click a submit button to refresh the model; (2) I/O transfer of the model, for example, to a Cloud backend for processing and pre-rendering; (3) complex model generation, transformation, and rendering computations, would introduce unnecessary friction in the modeling cycle. These concerns are shared with any textual-to-graphical modeling tool that—like BPMN Sketch Miner—is meant to be used in a live modeling context.

**Fig. 5:**
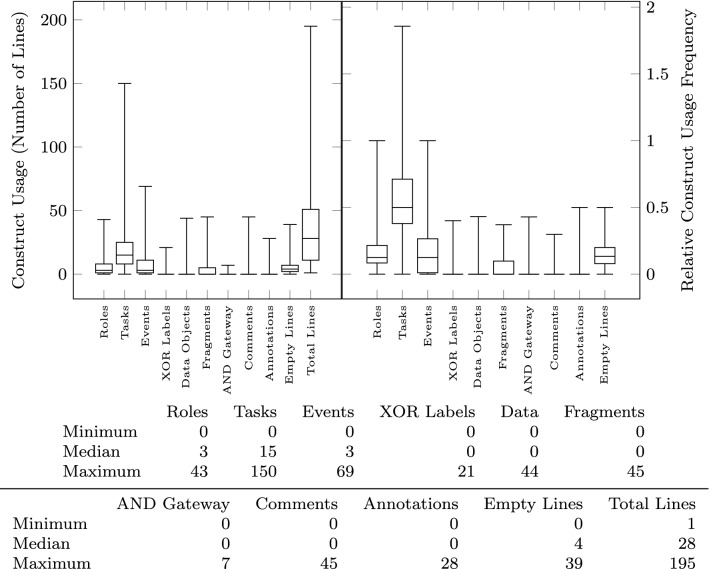
Absolute and relative textual DSL construct usage

**Fig. 6:**
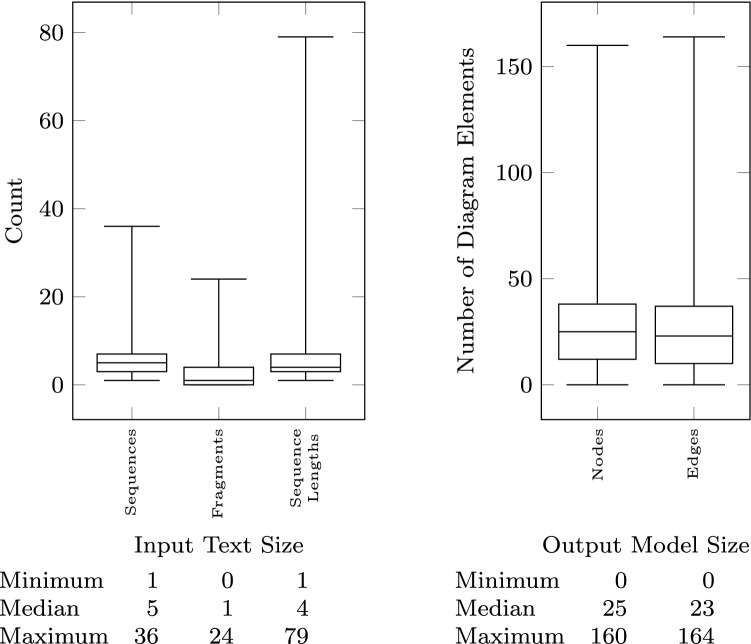
Text input and visual output model size

**Fig. 7:**
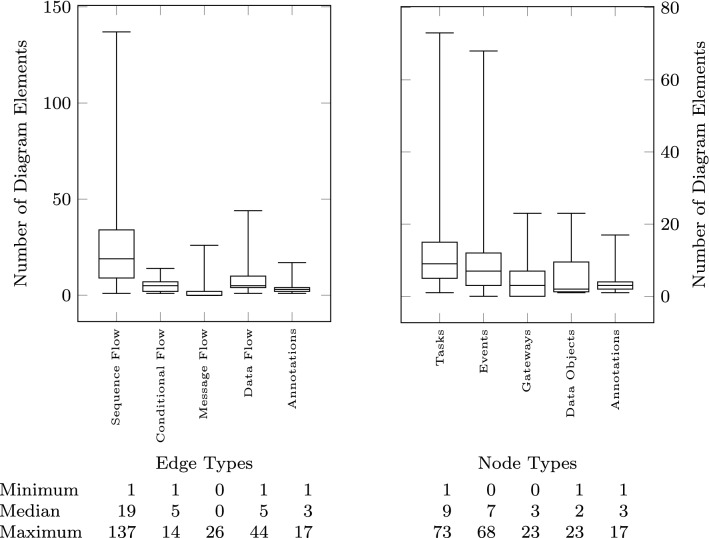
Output model: edge and node types

**Fig. 8:**
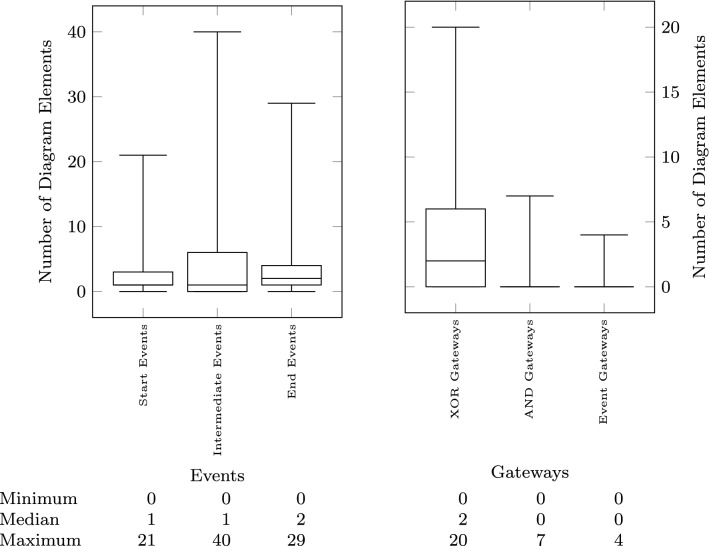
Output model: event and gateway types

In this performance evaluation, we do not only aim at determining whether the tool meets this requirement, but also to assess in depth which are the factors affecting its performance. In particular, we study the contribution of each architectural element of the tool’s pipeline (Fig. 2) to the execution time. This result can inform the designers of tools adopting a similar pipeline architecture about where to expect to encounter performance problems as well as to identify critical components for which an investment in performance optimization would have a positive return.

Other factors include the impact of the input size and input metamodel on the tool performance. Given the positioning of the tool as a BPMN sketching tool, we are interested to study how large and complex are the models that real-world users work with. Does the tool satisfy the live modeling requirement for all model sizes created by its users? While the workload characterization results we present are specific to the BPMN Sketch Miner domain-specific language, the same analysis would need to be performed for similar tools.

More in detail, our benchmark aims to answer the following specific research questions:

1. Is the performance of the tool sufficient to produce visual models as the user is typing their textual description?
2. Is there a bottleneck within the model generation pipeline?
3. How well does the tool scale to produce larger models?
4. To which extent does the tool performance depend on specific language constructs?

Answering RQ1 requires measuring the tool’s end-to-end performance but also obtaining a benchmark workload of models which are representative of real-world usage conditions. To detect bottlenecks (RQ2) in the pipeline, we profiled the execution so that we can identify which stage could require future performance optimization efforts. To observe the scalability of the tool (RQ3), we need first to determine how to measure the size of the textual input as well as the size of the resulting visual model. Regarding RQ4, we are interested to study the performance impact of the interplay between process mining and modeling for different BPMN constructs.

### Benchmark workload

Given the large variety of process models that can be represented, we selected a corpus of 1350 models which were exported by 396 different users of BPMN Sketch Miner during the year 2020. We expect users to export models at the end of their modeling session, so we can assume that the models have reached a good level of completeness. Also since the models are produced by end users, they should represent typical usage scenarios of the modeling tool in real-world conditions.

The criteria for including the models in the benchmark workload are:

- Completeness: The models were explicitly exported by the users at the end of their modeling session. We discarded models exported as part of tutorial sessions or short-lived sessions (less than 15 min).
- Coverage: The models are unique w.r.t. their input/output size metrics to sample the workload size parameter space as much as possible.

To characterize the models and how they make use of the textual domain-specific language, we present specific metrics counting how many lines and which types of lines are present (Fig. 5).

The size of the models is measured both in terms of their textual description (input to the pipeline) and in terms of the size of the visual model (output of the pipeline). In addition to the total number of lines (up to 200) shown in Fig. 5, we count how many sequences are present in the text (median of 5), how many lines are there in each sequence (sequence length—median of 4), and how many of those sequences are fragments (Fig. 6 right). To measure the size of the output, we first count how many nodes (up to 160) and edges (up to 164) are present in the resulting visual model (Fig. 6 left). We further classify the visual constructs found in the output models in Fig. 7 showing the number of sequence flow, conditional flow, message flow, data flow, and annotation associations, as well as the tasks, events, gateways, data objects, and annotations. Figure 8 further breaks down the events and gateways as they are inferred by the process mining algorithm.

To preserve users’ privacy, the exported models have been analyzed on the users’ own devices to measure their size according to the previous metrics. This information was later used to automatically synthesize the benchmark workload models with identical sizes but anonymized element labels [58].

### Performance metric

The performance is measured as the duration of one execution of the rendering pipeline. Within the modeling tool, this is triggered as soon as the user stops typing. Thus, we are interested to measure how long the user needs to wait before the visual model is refreshed to display the visualization obtained from the textual description.

We instrumented the model generation pipeline to record time measurements with the high-resolution timer provided by the performance.now standard JavaScript API.

### Testbed

The performance measurements have been obtained by running BPMN Sketch Miner version 1.17.4 on the Chrome Web browser version 88.0.4324.146 deployed on a 2018 Mac Book Pro (2.2 GHz Intel Core i7) running macOS 10.14.6.

It is out of the scope of this performance evaluation to use BPMN Sketch Miner as a benchmark to compare the performance of different Web browsers running on different OS/hardware configurations. Since BPMN Sketch Miner runs entirely on the Web browser, the performance experienced by the end users will clearly depend on their local runtime environment, but it will not be affected by their network conditions.

### Performance results

#### Total execution time

**Fig. 9:**
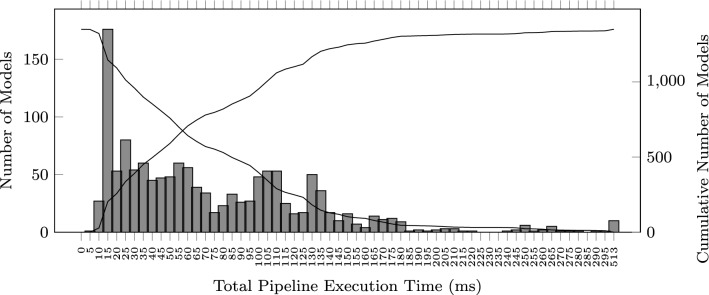
Total pipeline execution time histogram

Each model of the benchmark can be rendered in less than 513 ms, as shown in the histogram of Fig. 9. The median total execution time is 62.219 ms, and the mode is between 10 and 15 ms. On average, users editing the textual description of the models collected in the benchmark had to wait 77.255 ms before the corresponding visual representation was displayed.

#### Pipeline stages execution time

**Fig. 10:**
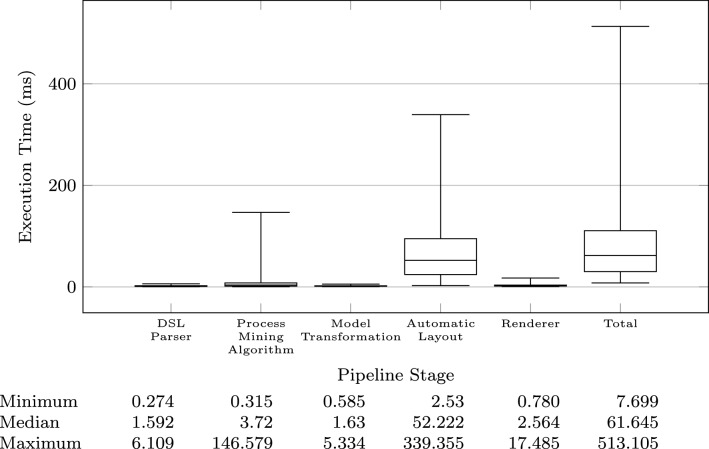
Execution time profile of the model generation and transformation pipeline

**Fig. 11:**
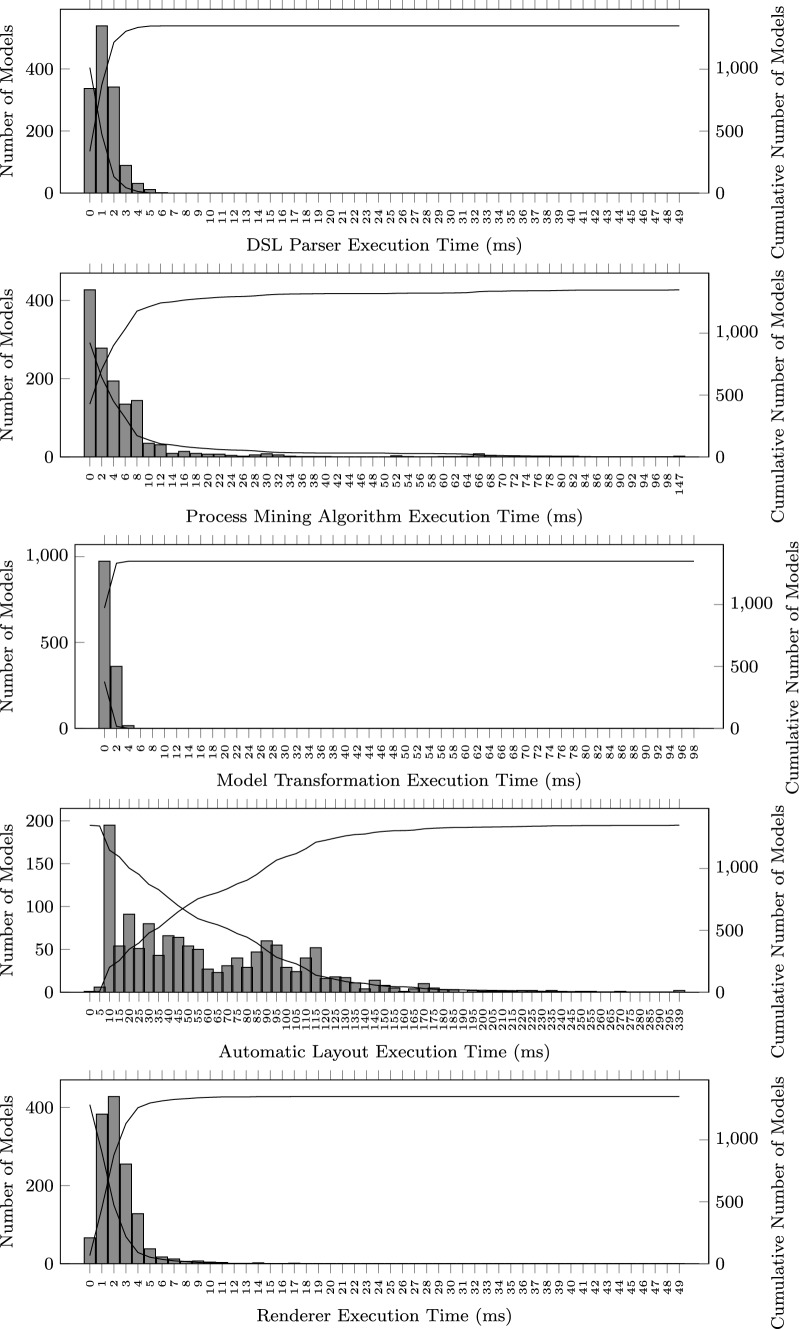
Execution time histograms for each pipeline stage

While running the benchmark, we have profiled the execution time of each pipeline stage. The results shown in Figs. 10 and 11 clearly indicate that the most time-consuming stage is the one running the automatic layout algorithm to position the model elements on the visual diagram.

As a reference, the median layout time is 56.864 ms, while 3.269 ms is the median time spent by the process mining algorithm. The other stages are faster with the following median times: 1.240 ms (parser), 1.929 (model transformation), and 3.140 ms (renderer).

The average execution times for each stage are: 1.755 ms (parser), 6.845 ms (mining algorithm), 1.715 ms (transformation), 64.151 ms (layout), and 2.787 ms (renderer).

#### Scalability

**Fig. 12:**
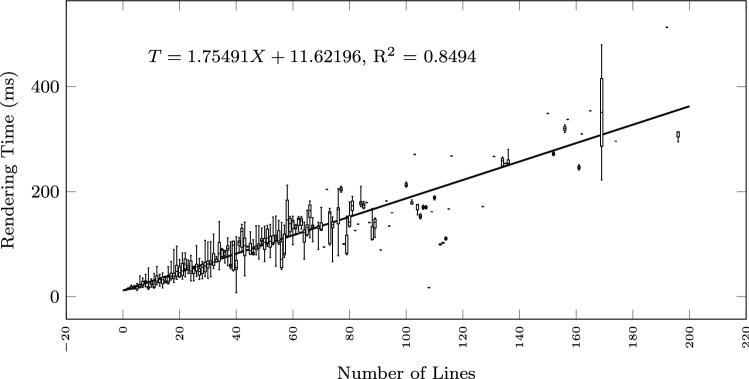
Model generation time versus input size: number of lines

**Fig. 13:**
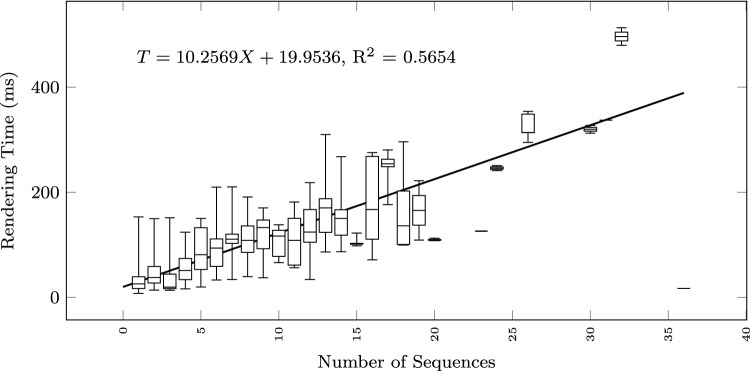
Model generation time versus input size: number of lines and sequences

**Fig. 14:**
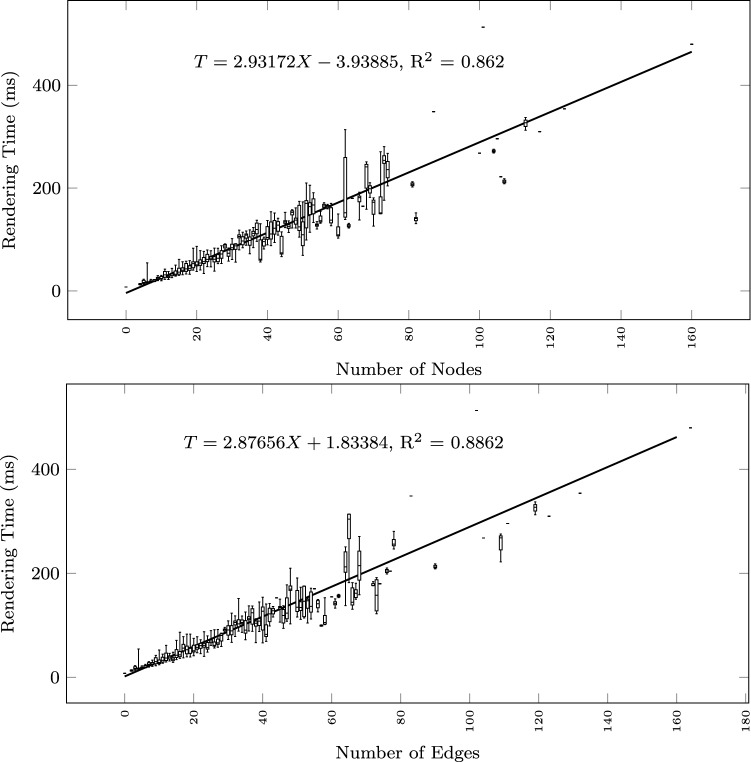
Model generation time versus output size: number of nodes and edges

**Fig. 15:**
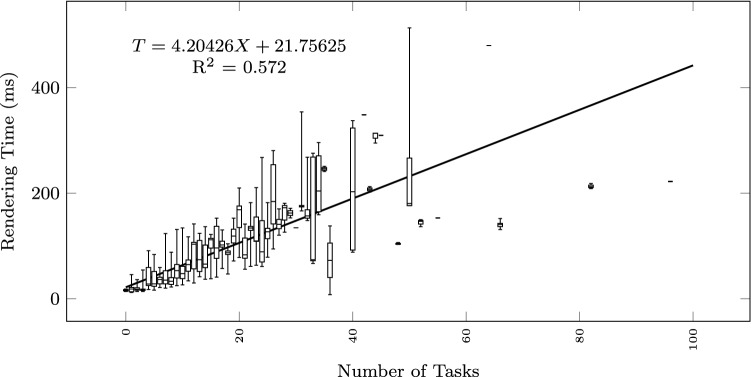
Model generation time versus output size: number of tasks

**Fig. 16:**
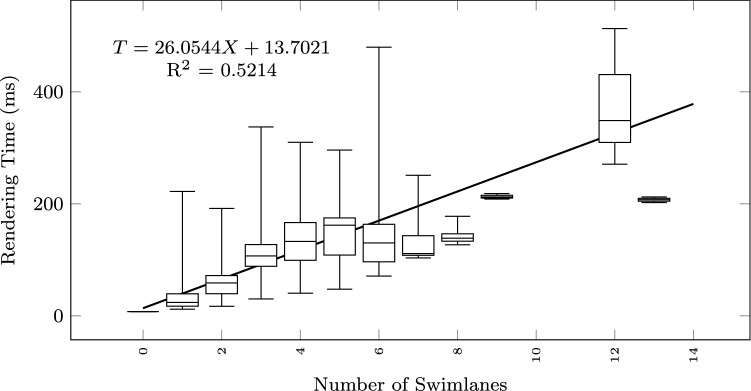
Model generation time versus output size: number of swimlanes

**Fig. 17:**
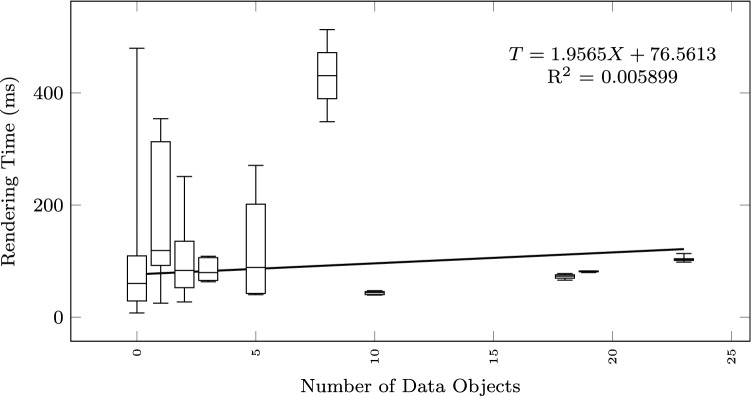
Model generation time versus output size: number of data objects

**Fig. 18:**
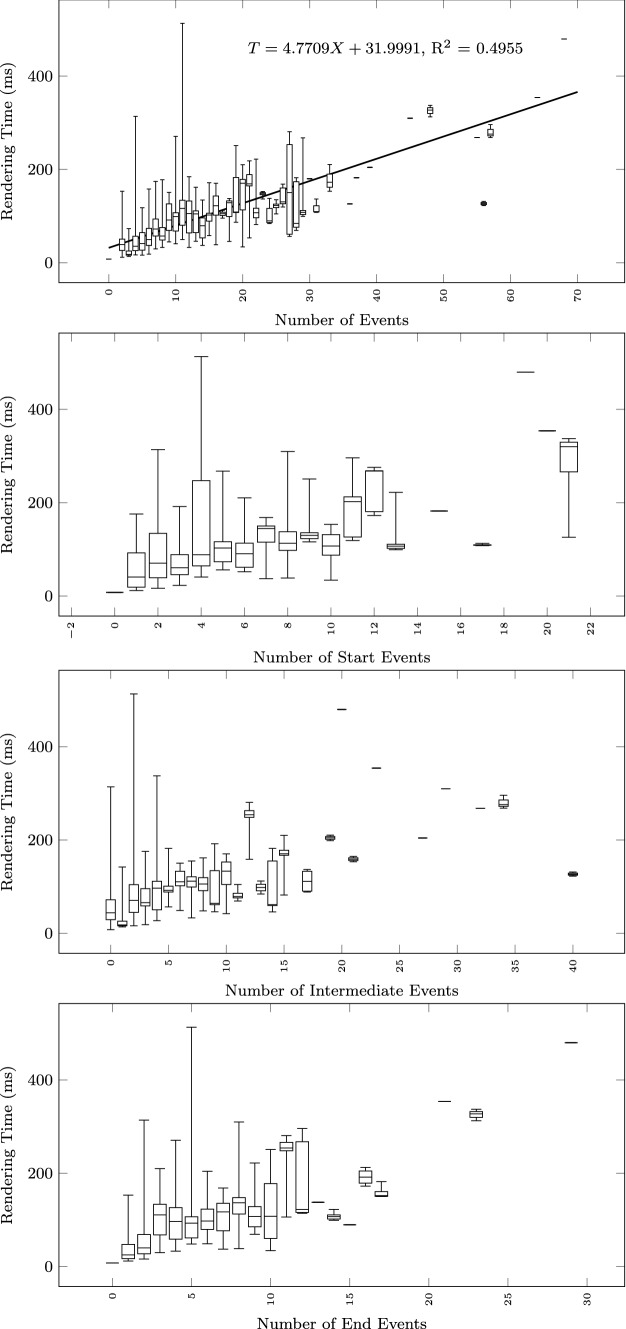
Model generation time versus output size: number of events

**Fig. 19:**
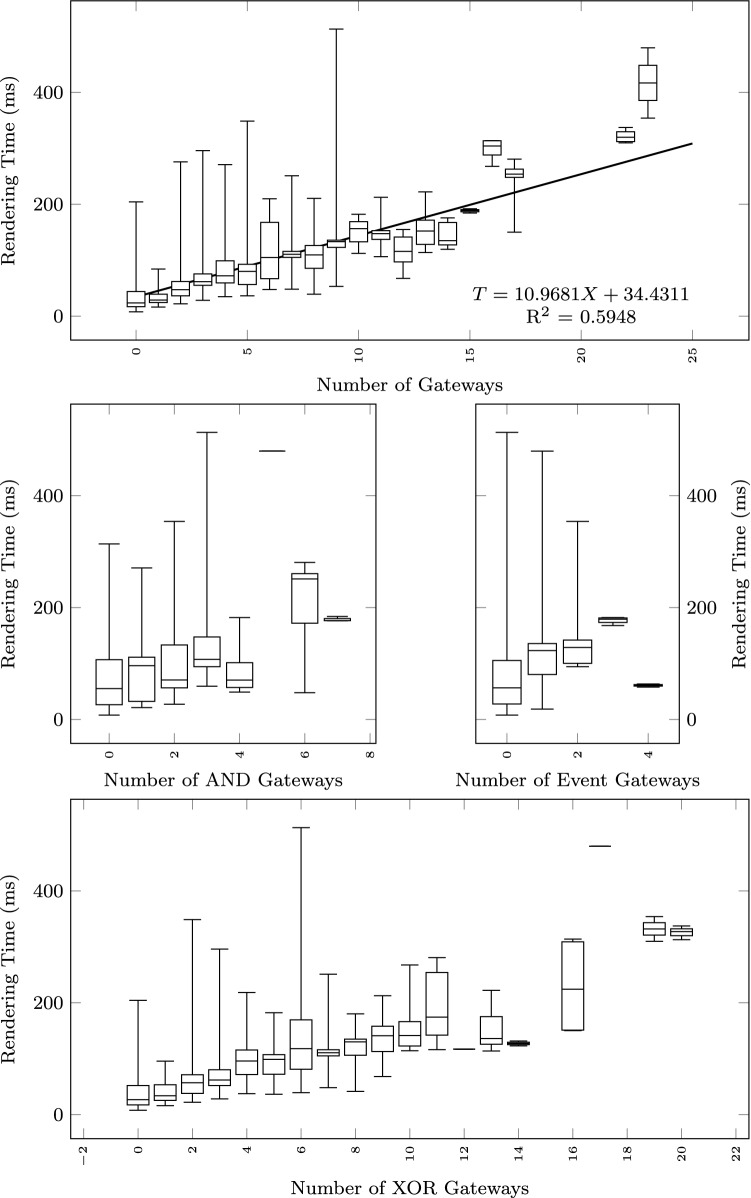
Model generation time versus output size: number of gateways

**Fig. 20:**
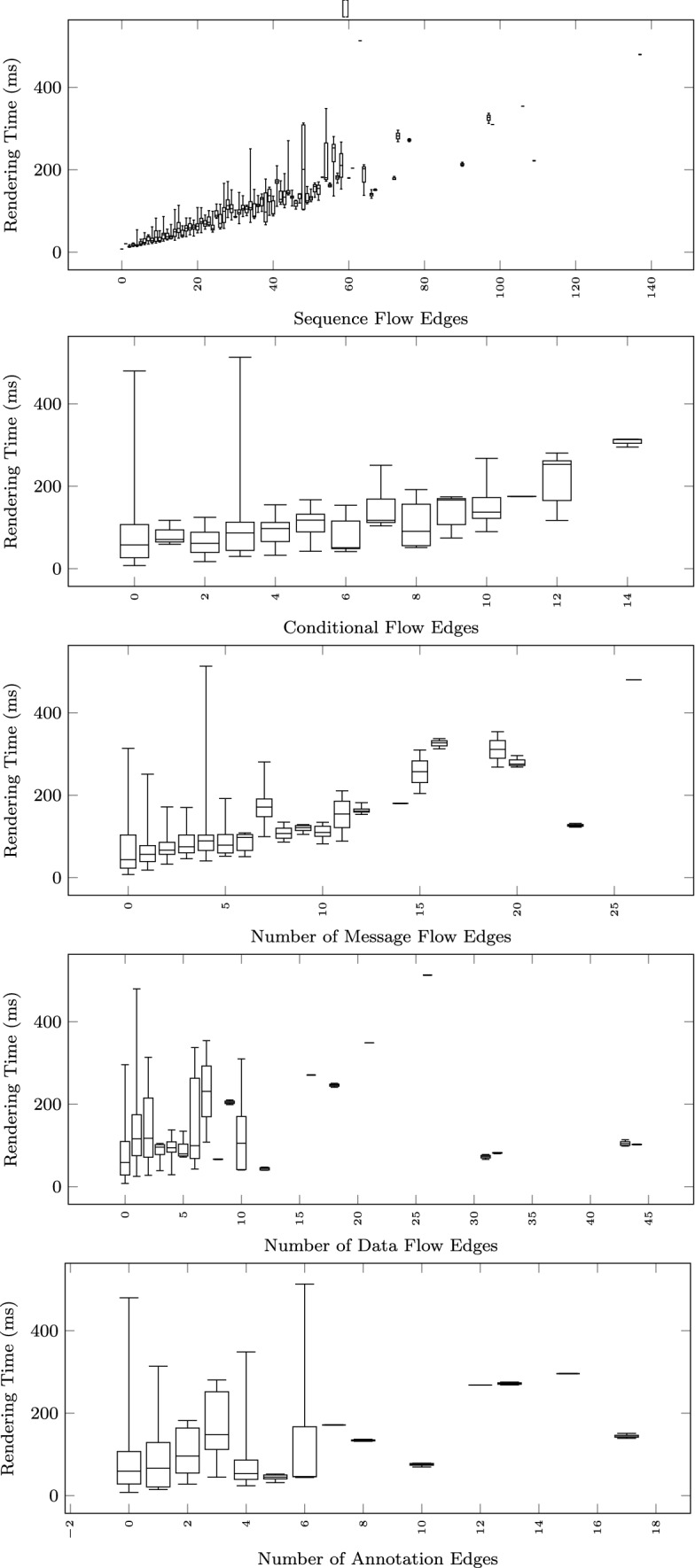
Model generation time versus output size: edge types

To study how the execution time grows with the size of the input text and the output diagram, we observe the total execution time as a function of different size metrics: considering the input size: number of lines of the input textual description (Fig. 12), and number of sequences found in the textual description (Fig. 13); considering the output size: the number of nodes and edges of the BPMN diagram (Fig. 14), the number of tasks (Fig. 15), swimlanes (Fig. 16), data objects (Fig. 17), events (Fig. 18), gateways (Fig. 19), and different types of flow edges (Fig. 20).

For every value of the size metric, we display in “Appendix” a boxplot illustrating the time measurement distribution.

Given the user-generated input, most samples are obtained with low size values. For some metrics, we include a linear regression model attempting to interpolate the measurements across the entire range of values. We also use it to assess whether the execution time can be considered linearly dependent on the input and output size.

#### Performance impact of BPMN constructs

To study the impact of the mix of modeling constructs found in a model on the pipeline execution time, we provide linear regression models for most of the previous charts. We have also fitted a multiple linear regression using a set of independent output size metrics (Table. 5).

While all independent variables have a significant impact on the execution time, the strongest positive contribution is provided by the number of swimlanes, followed by the number of edges and gateways. The presence of other types of nodes (tasks, events, data objects, and annotations) appears to reduce the execution time.

### Discussion

#### RQ1. Is the performance sufficient for live modeling?

In the worst case, the delay does not grow above 513 ms. For most models, the time stays under 50ms. This is fast enough [4, 49].

**Table 5:** Multiple linear regression model

	Dependent variable
	Time
Number of Swimlanes	9.426$$^{***}$$
	(0.347)
Number of Edges	3.235$$^{***}$$
	(0.124)
Number of Gateways	1.220$$^{***}$$
	(0.229)
Number of Annotations	− 0.739$$^{***}$$
	(0.255)
Number of Tasks	− 1.362$$^{***}$$
	(0.116)
Number of Events	− 1.679$$^{***}$$
	(0.140)
Number of Data Objects	− 2.439$$^{***}$$
	(0.286)
Constant	− 1.102
	(0.745)
Observations	1,350
$R^{2}$	0.944
Adjusted $R^{2}$	0.943
Residual Std. Error	13.911 ($df = 1342$)
F Statistic	3211.260$$^{***}$$ ($$df = 7$$; 1342)

$$^{*}p<0.1$$; $$^{**}p<0.05$$; $$^{***}p<0.01$$

This result confirms our design decision to implement BPMN Sketch Miner to run entirely in a Web browser to avoid the server-side rendering round trip delay of tools such as PlantText, which require users to explicitly submit their textual description to be rendered. With BPMN Sketch Miner, by the time users have stopped typing and are considering whether the visual diagram should be refreshed, the result of the rendering pipeline is already displayed.

#### RQ2. Is there a bottleneck in the pipeline?

Yes, the automatic layout algorithm is the most expensive stage.

While generating the model using a process mining algorithm simplifies the modeling task of users—who, for example, do not have to worry about creating the correct control flow graph structure or explicitly selecting among start, intermediate, or end events—it also does not appear to introduce significant overhead in the process generation pipeline, especially when compared with the automatic layout, which also helps to reduce the tedious manual drag and drop effort required by many manual visual modeling tools.

The pipeline profiling results help us to focus future optimization efforts on the layout stage, which will be critical to support additional language features such as sub-processes.

#### RQ3. How well does it scale to larger models?

As shown by the results collected in Figs. 12, 13, 14, 15, 16, 17, 18, 19 and 20, according to many different size metrics, the pipeline execution time appears to scale linearly with the size of the input and the output. Within the operating range of model sizes covered by the benchmark, the tool performance remains within acceptable bounds.

The original motivation for BPMN Sketch Miner was to rapidly support sketching activities of simple process models. Can a model with hundreds of tasks still be considered a sketch? Actual usage scenarios with such large models may require us to reconsider some of the basic assumptions behind the tool’s user interface design and its live editing cycle, which was not originally intended to be used with textual descriptions beyond one hundred lines.

As users venture beyond the limits of the tool operating range, it will become more difficult to maintain the same live modeling experience with larger and larger models. The slowest performance observed of 513ms is already close to what some users may have perceived as a noticeable lag [50]. One simple solution would be to add a refresh button and give manual control to the user about when the pipeline should be relaunched. Another more technically challenging approach would be to introduce some dynamic algorithms which can incrementally update the model based on detecting which changes have been applied to the textual description [23].

#### RQ4. What is the impact of specific language constructs on performance?

The multiple linear regression model of Table 5 highlights the swimlanes as the construct with the most prominent impact on the execution time. This can be explained by how swimlanes constrain the layout algorithm when placing the diagram elements and by the need to reshuffle swimlanes into separate pools in case of message flow exchanges between them.

The negative weights of some constructs can be explained by the fact that the model obtained from the process mining algorithm is always a connected graph. Thus, for every model element (such as tasks or events, or data objects) there is always at least one edge present in the diagram. In absolute terms, Edges have a cost that is higher than all of the other types of nodes.

### Threats to validity

The models exported by users have a median size of 25 nodes and 23 edges and have been described with 28 lines of text (median). Compared to the largest modes in the workload having up to 160 nodes and 164 edges (described using up to 195 lines of text), the distribution is skewed toward smaller models. The linear scalability claim is valid only within the given workload range, which reflects the current operating range of the tool.

Given the fully automatic, privacy-preserving data collection process we followed to obtain the workload models, it is not possible to assess whether the models were exported by occasional users who just tested the tool as opposed to models representing actual purposeful usage. The filtering criteria we have applied to select the models (e.g., discarding models exported during tutorial or short-lived sessions) are intended to mitigate this threat.

As with every benchmark, the actual performance ultimately experienced by the users will depend on their own Web browser, OS, and hardware stack configuration.

## Related work

### Empirical studies comparing graphical versus textual representations

Researchers have been long discussing whether textual or graphical model representations are better [19, 23, 26, 34, 46, 48, 54]. Many empirical studies comparing graphical versus textual representations have been carried out in the past concerning, e.g., COBOL data structure documentation [38], requirement models [40, 57], UML model maintenance [34, 46], or declarative [24] and imperative [6] business process models.

Sharafi et al. [57] performed an eye-tracking experiment with 28 participants to study their efficiency and accuracy with requirement comprehension tasks. While no impact on accuracy was observed, the subjects were significantly slower when working with the TROPOS graphical representation. In the same domain of requirement modeling and analysis, a recent controlled experiment with 38 student participants compared the textual and graphical versions of iStar [40] focusing on modeling tasks, with no significant differences on the resulting model size and accuracy when using the versions of iStar. However, it was found that “participants prefer to model graphically.” A similar result was obtained by Melia et al. [46] with a pilot study involving 86 students at the University of Alicante comparing UML versus a textual OO language syntax. The authors highlighted the main benefits of each notation type and performed an empirical comparison of their usability. They analyzed the impact the two modalities have on the efficiency, effectiveness, and satisfaction of novice programmers while performing maintenance tasks such as model understanding, error detection and correction. Their quasi-experiment using the OOH4RIA tool has shown that the novice programmers discovered more errors and were more efficient in fixing them when using the textual notation, but expressed preference for the graphical notation.

Jolak et al. [34] conducted an experiment with 240 software students from four different universities to study the impact of the type of representation (textual vs. graphical) on user’s ability to explain, understand, recall, and actively communicate knowledge. They used UML class diagrams for the graphical representation. They discovered that the graphical representation has a positive effect on the explaining and understanding ability, but with no statistical significance, while a statistically significant advantage of graphical representation over textual representation is noticed for the recall ability. Also, a statistically significant effect was noticed regarding the ability to actively communicate knowledge. Namely, it has been observed that the graphical representation fosters more active discussion than the textual representation and has a positive effect on the creative conflict discussions while requiring less conversation management effort.

Closer to the domain of our work, Haisjack and Zugal [24] investigated the differences between graphical and textual declarative process models. The main result obtained with a sense-making study involving 9 subjects was that “the graphical representation is advantageous in terms of errors, duration, and mental effort.” Rodrigues et al. [6] described an experiment to compare the clarity of work instructions represented as text or as BPMN process models, with no differences in understandability only for non-experts. Instead, increased process understanding was found for experienced users using BPMN models.

### From text to visual process models

The previous studies have compared models presented alternatively with textual versus graphical representations and evaluated which modality was found easier to understand by the subjects. In our work, we see the two representations as complementary. In particular, we advocate writing the content of the model using text and simultaneously reading the content of the model from the corresponding visual notation. In [30], we have introduced the idea of obtaining BPMN models by means of mining traces described using a textual DSL. We presented an environment for analysts and process participants where they can rapidly sketch business process models as they discuss them using natural language during structured interviews. The approach supported only a very small number of BPMN constructs (start/end event, XOR gateway, task, and sequence flow BPMN constructs) and lacked the modeling annotations necessary to distinguish different types of tasks and events, pools, message flow, etc. The tool presented in [31], as well as in this article, is still based on process mining [65], and as such provides access to the core constructs of the BPMN notation without requiring an explicit description of the process control flow, e.g., when using the PlantUML textual syntax for activity diagrams. On the other hand, it provides a much more expressive DSL compared to the one we presented in [30] by combining the process mining with textual modeling.

Using textual DSL for process modeling is not a new idea. Starting from the same motivations as ours, the authors of [29] proposed a textual DSL for describing S-BPM [14] processes. However, unlike the BPMN Sketch Miner’s textual DSL, the S-BPM is designed for explicitly declaring the whole model’s structure textually. T-Square [55] is another declarative textual DSL for rapid processes description by specifying the tasks and the branching conditions. This DSL is incorporated in NOVA [43] which is an Eclipse-based editor which enables modeling workflows graphically based on the Compensable Workflow Modeling Language (CWML). While the goal of our textual DSL is generating a visual BPMN compliant artifact that can be exported in different formats, including the BPMN XML format, the goal of T-Square is to generate executable workflows from textual specifications, by means of a model transformation using Xtend.

BPMN Sketch Miner’s goal of speeding up the initial model construction phase is also shared with the Rapid Business Process Discovery (R-BPD) tool [21], whose solution combines both text-to-model and model-to-model transformations, which can extract models from any textual resource discovered in an enterprise repository and also foster the reuse of existing models. However, none of the above-stated textual DSLs targets BPMN as a modeling language. To the best of our knowledge, the only existing work on textual modeling for BPMN is PlantBPMN [16], which is a textual DSL created using the Xtext [10] framework, supported by an Eclipse-based editing tool. A model transformation between the metamodels created by the BPMN textual DSL and the BPMN XML Schema is implemented. The DSL presented in this article is more abstract compared to PlantBPMN, as BPMN Sketch Miner uses process mining to infer the presence of many constructs (e.g., exclusive vs. event-based gateways, pools vs. swimlanes, start vs. intermediate vs. end events, etc.—see Table 1 for details) which need to be explicitly detailed in PlantBPMN.

While our text to visual model transformation is motivated by streamlining the process modeling workshop feedback cycle, there are many tools targeting the reconstruction of models starting from preexisting documentation written in natural language [2]. Concerning business process models, text highlighting [1] augmented with natural language processing (NLP) [41] has been proposed as a technique to guide the transition from informal text-based process descriptions to formal declarative process models. Honkisz et al. [28] proposed to use an intermediate spreadsheet-like tabular representation between the raw input text and the final BPMN model. Ferreira et al. [13] proposed a semi-automatic, rule-based approach to identify process elements in natural language texts featuring 33 mapping rules. Friedrich et al. [18] presented a work which aimed at generating BPMN models using a set of natural language processing (NLP) techniques. Their proposed technique was evaluated using 47 texts in natural language. The tool could generate accurate process models for 77% of the texts. This approach has been successfully applied to reconstruct processes within specific domains (e.g., archeological excavation methods [9]). More recently, similar work by van der Aa et al. [61] shows how to obtain declarative process models from natural language. However, our approach supports business analysts during requirements gathering interviews where the process is being described for the first time or its initial sketch needs to be agreed upon with process participants, or when written process documentation is not available, which is the main prerequisite for using the above-discussed natural language processing approaches. The DSL designed for the BPMN Sketch Miner is meant to be close, as much as possible, to natural language, in order to ease the user’s task of memorizing the concrete syntax of the language. Based on the textual model that the user introduces, the visual BPMN model is automatically generated in real time.

The goal behind the liveness [59] of the BPMN Sketch Miner is to minimize the latency between a modeling action applied to the textual description of the process and seeing its effect on the resulting diagram. Such need of “instant feedback and shared understanding” of a business process between the business analyst and the domain experts has also been recognized by Grosskopf et al. [42]. Contrary to our approach, they use tangible BPMN elements (t.BPM) to be moved around by domain experts while physically building the visual business process model on a table. They have found that t.BPM allows domain experts to identify the need for model corrections faster, due to the gradual building up of the visual model, and it motivates them to think more about their process. Dixit et al. [7] take a different approach to include the domain expert in the process discovery by providing suggestions for the next possible constructs to be added to the model based on the probabilities discovered with process mining algorithms. This approach is not applicable for processes which lack event logs.

### Model transformation performance benchmarking

Performance benchmarks are also present within the MDE community, where model transformations play a critical role. In [64], the authors evaluated and compared the performance of three of the most used transformation languages in MDE: ATL, QVT Operational Mapping, and QVT Relations. The experiments compared the execution duration of two examples of model transformations implemented using each of these languages. The goal was to identify all the factors that influence the performance of the transformation engines under test, in order to provide to their users the possibility to reach higher performances by tuning these parameters. While the authors of [64] proposed a performance evaluation tool specific for ATL and QVT, MONDO-SAM [32] provides an extensible MDE benchmarking framework, to measure the scalability of different tools against specific complexity metrics.

Similar to our performance evaluation approach, both [64] and [32] frameworks take as inputs real-world model instances. He, Zhang, Hu, Ma, and Shao [25] instead propose an algorithm for the efficient random generation of correct models, which can be used as benchmark workload when user-provided models are not available.

### Transforming process models back into natural language

A survey on the challenges and opportunities of applying NLP in BPM has been presented at the computational linguistics conference [60]. The authors also covered the opposite practice of text generation from BPMN models. Such visual-to-text transformation can be motivated by the finding that graphical notations require formal training to be correctly understood [54], while textual representations using natural language convey their meaning to both professionals with BPM background as well as to business stakeholders from a wider business setting [52].

Leopold, Mendling and Polyvyanyy [39] developed an automatic approach for generating natural language texts from business process models “combining natural language analysis, graph decomposition techniques and a linguistic framework for flexible text generation.” Process-To-Text [15] instead is a framework for the quantitative description of processes in natural language, which can enrich the natural language description with quantitative information about past process execution extracted by mining event logs.

## Conclusion

Most of the existing BPMN modeling editors are graphical editors where users are required to drag, drop, and connect visual constructs. This frequently results in BPMN models being generated by the business analyst as a second step after a requirements elicitation workshop, which contributes to a lengthier feedback cycle as a subsequent meeting is needed to discuss whether the generated model reflects accurately what has been discussed with the stakeholders during the workshop. To overcome this, we have developed the BPMN Sketch Miner to support live process modeling using a constrained natural language textual input which mimics notes taking during a requirements elicitation meeting. This article presents the design and evaluation of the BPMN Sketch Miner in terms of its usability, learnability, expressiveness, and performance, extending the previous MODELS 2020 publication [31].

The design of the textual BPMN implemented in the tool attempts to combine together modeling (prescribing what the process does) and mining (describing what the process does) by using constrained natural language. The results from our evaluation show that the trade-off between expressiveness and usability is well balanced. The survey respondents mostly found the BPMN expressiveness as sufficient, as also indicated by the mix of constructs present in the workload model collection. Regarding the performance evaluation, the tool complies with the rapid sketching and live modeling requirement we set in its design as, across the entire benchmark, the rendering time remains below 513ms. More in detail, comparing the execution times of the different phases of the model generation and transformation pipeline revealed that the automatic layout is the most time-consuming stage. We have also calculated, for each BPMN construct, the different correlations between the execution time and the number of times a specific construct is found in the BPMN model. Overall, the models’ rendering time is mostly impacted by the number of swimlanes and the number of edges in the model.

All in all, we demonstrate the feasibility of using a Web browser as a live BPMN modeling environment featuring a low-latency text-to-visual model transformation. The proposed pipeline architecture is generally applicable to similar tools, which feature as part of their input both instance examples from which the model should be inferred and the explicit description of the model. The automatic layout may turn out to be a bottleneck in other text-to-visual tools, whose input defines the model element graph topology but not exactly where to place the visual elements on the diagram.

## Future work

As future work, we plan to continue extending the set of BPMN constructs supported by the textual DSL as prioritized by the feedback of our user community. As this may put additional pressure on the automatic layout algorithm, we may need to introduce a dynamic [27] variant of the pipeline, which—for some frequent types of changes applied to the input—may produce incremental model editing operations to be locally applied to the output, thus avoiding to refresh the entire model after every keystroke. This will start to pave the way toward supporting two-way, text-to-visual and visual-to-text, synchronization.

Regarding scalability and impact of language constructs, we found mostly linear dependencies of run time w.r.t. complexity measures of the input. We do not necessarily expect that the process mining and layout algorithms exhibit a similar behavior for larger workloads. As the tool is being used more widely, it may happen that users will not only sketch small process models but attempt to create yet bigger models. We plan to extend this study with also bigger examples, perhaps created artificially with certain characteristics, to assess whether the linear-time complexity result also holds for larger models. This will be key to determine where the threshold lies for live-modeling to stop being an option. We are also planning to introduce in the next release of the tool the option for users to control the automatic live text-to-graphical synchronization. This way, users themselves can determine on a individual basis whether the delay is acceptable or not, both depending on their local hardware capacity and the size and complexity of their models.

While this article focused on the performance of the tool itself, more work is needed to assess the performance and productivity of its users. For example, we are interested to recruit users interested in participating in empirical studies to observe: (1) how long it takes to obtain the first version of a model which can be subjected to a round of feedback with the stakeholders; (2) how easy is it to quickly apply suggestions for improvements while refining the models as opposed to traditional visual environments; (3) what is the end-user perceived performance of the tool within their usual modeling activities; and (4) what difficulties may arise when using the tool with larger and more complex models as opposed to initial model sketches. We are also planning to provide collaborative editing capabilities of the text editor which could further help increasing the efficiency of process elicitation workshops or interviews.

## Appendix: model generation time versus model size

In this appendix, we collected the plots showing the relationship between the total time required to obtain a model and the size of the model in terms of many different metrics. They provide evidence for both RQ3 (scalability) and RQ4 (impact of specific BPMN constructs) as presented in Sect. 4.4.

## Footnotes

1 https://plantuml.com/.

2 https://www.planttext.com/.

3 https://app.zenuml.com/.

4 https://www.websequencediagrams.com/.

5 https://textografo.com/.

6 https://www.bpmn-sketch-miner.ai.

7 This article includes and extends the work that the authors have presented at the 23rd International Conference on Model-Driven Engineering Languages and Systems (MODELS) under the title “From Text to Visual BPMN Process Models: Design and Evaluation” [31].

8 https://github.com/dagrejs/dagre-d3.
